# A multicenter chart review of patient characteristics, treatment, and outcomes in hereditary angioedema: unmet need for more effective long-term prophylaxis

**DOI:** 10.1186/s13223-023-00795-2

**Published:** 2023-05-29

**Authors:** Joan Mendivil, Maral DerSarkissian, Aleena Banerji, Lavanya Diwakar, Constance H. Katelaris, Paul K. Keith, Harold Kim, Gina Lacuesta, Markus Magerl, Charlotte Slade, William B. Smith, Zia Choudhry, Angela Simon, Sujata P. Sarda, Paula J. Busse

**Affiliations:** 1 Takeda Pharmaceuticals International AG, Zurich, Switzerland; 2 Analysis Group, Inc., Boston, MA USA; 3 Division of Rheumatology, Allergy and Immunology, Department of Medicine, Massachusetts General Hospital, Harvard Medical School, Boston, MA USA; 4 Department of Immunology, University Hospitals of North Midlands, Stoke-on-Trent, UK; 5 Campbelltown Hospital, Western Sydney University, Campbelltown, NSW Australia; 6 McMaster University, Hamilton, ON Canada; 7 Department of Medicine, Western University, London, ON Canada; 8 Halifax Allergy and Asthma Associates, Halifax, NS Canada; 9 Institute of Allergology, Charité - Universitätsmedizin Berlin, Freie Universität Berlin and Humboldt-Universität Zu Berlin, Berlin, Germany; 10 Fraunhofer Institute for Translational Medicine and Pharmacology ITMP, Immunology and Allergology, Berlin, Germany; 11 Department of Clinical Immunology and Allergy, Royal Melbourne Hospital, Melbourne, VIC Australia; 12 Department of Clinical Immunology and Allergy, Royal Adelaide Hospital, Adelaide, SA Australia; 13 Takeda Development Center Americas, Lexington, MA USA; 14 Division of Allergy and Clinical Immunology, Icahn School of Medicine at Mount Sinai, New York, NY USA

**Keywords:** Hereditary angioedema, C1 inhibitor deficiency, Long-term prophylaxis, On-demand treatment, Androgens, Retrospective chart review, Healthcare resource utilization

## Abstract

**Background:**

Hereditary angioedema (HAE) is a rare disease characterized by unpredictable, recurring subcutaneous or submucosal swelling. Without effective therapy, HAE can negatively impact patients’ quality of life. Management of HAE includes on-demand treatment of attacks and short- and long-term prophylaxis (LTP) to prevent attacks. Newer therapies may be more tolerable and effective in managing HAE; however, therapies such as androgens are still widely used in some countries owing to their relative ease of access and adequate disease control for some patients. This study evaluated the characteristics, treatment patterns, clinical outcomes, and healthcare resource utilization of a multinational cohort of patients with HAE, with a focus on understanding reasons for recommending or discontinuing available therapies.

**Methods:**

A retrospective chart review was conducted at 12 centers in six countries and included data from patients with HAE type 1 or 2 who were ≥ 12 years of age at their first clinical visit. The relationship between LTP use and attack rates was evaluated using a multivariable Poisson regression model. Data were collected between March 2018 and July 2019.

**Results:**

Data from 225 patients were collected (62.7% female, 86.2% White, 90.2% type 1); 64.4% of patients had their first HAE-related visit to the center prior to or during 2014. Treatment patterns varied between countries. Overall, 85.8% of patients were prescribed on-demand treatment and 53.8% were prescribed LTP, most commonly the androgen danazol (53.7% of patients who used LTP). Plasma-derived C1 inhibitor (Cinryze^®^) was used by 29.8% of patients for LTP. Patients who received LTP had a significantly lower rate of HAE attacks than patients who did not receive any LTP (incidence rate ratio (95% confidence interval) 0.90 (0.84–0.96)). Androgens were the most commonly discontinued therapy (51.3%), with low tolerability cited as the most frequent reason for discontinuation (50.0%).

**Conclusions:**

Overall, findings from this study support the use of LTP in the prevention of HAE attacks; a lower rate of attacks was observed with LTP compared with no LTP. However, the type of LTP used varied between countries, with tolerability and accessibility to specific treatments playing important roles in management decision-making.

**Supplementary Information:**

The online version contains supplementary material available at 10.1186/s13223-023-00795-2.

## Background

Hereditary angioedema (HAE) is a rare, severely debilitating, autosomal dominant disorder that causes unpredictable attacks of subcutaneous or submucosal swelling [1]. HAE prevalence is estimated at 1:50,000 worldwide, however, there is considerable variation between countries [2–5]. C1 inhibitor (C1-INH) deficiency is the most common form of HAE and is subdivided into two types: type 1 affects approximately 85% of patients and is characterized by low levels of functional C1-INH; type 2 affects approximately 15% of patients and is characterized by normal or elevated levels but reduced functional activity of C1-INH [3].

HAE types 1 and 2 are managed similarly. This includes on-demand treatment (ODT) of attacks, short-term prophylaxis (STP) before anticipated triggers such as surgical or invasive dental procedures, and long-term prophylaxis (LTP) to prevent recurrent attacks [2, 6]. All patients with HAE, particularly those with symptoms that impact their quality of life, are encouraged to discuss the benefits of LTP with their physician [2].

Attenuated androgens such as danazol, stanozolol, and oxandrolone have been used for LTP and have varying effectiveness [7]. They are still prescribed in some countries and regions, primarily due to accessibility, cost effectiveness, and the ease of oral administration; in addition, some patients report adequate effectiveness and tolerability [7]. However, limitations of androgen therapy include substantial adverse events such as weight gain, muscle cramps, seborrhea, acne, and virilization in women [8, 9]. Furthermore, androgens are contraindicated during pregnancy and should be avoided in pediatrics [6, 10]. Use of androgens has also been shown to increase the risk of comorbidities such as anxiety/depression, muscle cramps, obesity, and hyperlipidemia [11].

Recent advancements in treatment options for LTP that are highly effective and well tolerated include plasma-derived C1-INH concentrates and plasma kallikrein inhibitors [12]. C1-INH concentrates that are approved for LTP (Cinryze^®^ and Haegarda^®^) markedly reduce HAE attack rates [13, 14]. However, a major limitation of these therapies is the need for frequent administration (every 3–4 days) by intravenous or subcutaneous injection. More recently, approved kallikrein inhibitors (lanadelumab, a monoclonal antibody administered subcutaneously every 2 or 4 weeks [15], and berotralstat, a daily oral inhibitor [16]), have demonstrated efficacy in preventing attacks. Despite the benefits of these modern treatments, their availability is limited in many countries, and high costs place a heavy burden on healthcare systems [17].

A multicenter chart review study was conducted to evaluate clinical characteristics of patients, treatment patterns, outcomes, and healthcare resource utilization (HRU) in international real-world clinical practice. This study provides a greater understanding of reasons for the recommendation or discontinuation of treatments, thereby highlighting current unmet needs and disease burden in HAE.

## Methods

### Study design

A multicenter, retrospective, longitudinal chart review study was conducted at 12 clinical centers specializing in HAE care across six developed countries (Australia, Canada, Germany, Italy, the United Kingdom, and the United States). Data were collected between March 2018 and July 2019. Staff at each clinical center extracted data from patients’ charts (i.e., medical records) using a uniform electronic case report form. The case report form collected information on patient demographic and clinical characteristics, diagnostic journey, clinical HAE outcomes, treatment patterns, and HRU. The date of the first HAE-related visit at the participating clinical center was considered the index date (Additional file 1: Fig. S1). The baseline period was defined as the 6-month period before the index date. The observation period began on the index date and ended with the last clinical visit, enrollment in a clinical trial for an investigational HAE treatment, death, or end of study (whichever occurred first). Patients had at least two HAE-related visits to the clinical center (one index visit and at least one visit during the follow-up period). All patients who met study eligibility criteria at each clinical site were included.

### Eligibility criteria

Patients had a physician-documented diagnosis of HAE type 1 or 2 and were ≥ 12 years of age at index. To capture the current treatment landscape, ≥ 1 documented HAE-related visit at the center must have occurred within the 2 years preceding chart abstraction. Patients were excluded if they had a diagnosis of HAE with normal C1-INH or acquired angioedema or if they had only one visit to the center.

### Data analysis

Descriptive statistics were used to summarize the demographic and clinical characteristics of patients during the baseline period, patient treatment patterns (i.e., ODT, STP, LTP), and attack characteristics. Attack severity was based on the patient’s ability to perform regular daily activities [18]. For each patient, the HAE attack rate was calculated by dividing the number of attacks by the total observation time over which information on frequency of HAE attacks was available. The rate was then standardized to a denominator of 1 year to obtain an annual rate. LTP treatment patterns were evaluated at the treatment period level instead of at the patient level, as patients could have had multiple treatments during follow-up. The relationship between LTP use and HAE attack rate was evaluated using a multivariable Poisson regression model, adjusting for selected baseline patient demographic and clinical characteristics.

## Results

### Patient demographics and attack characteristics

**Fig. 1:**
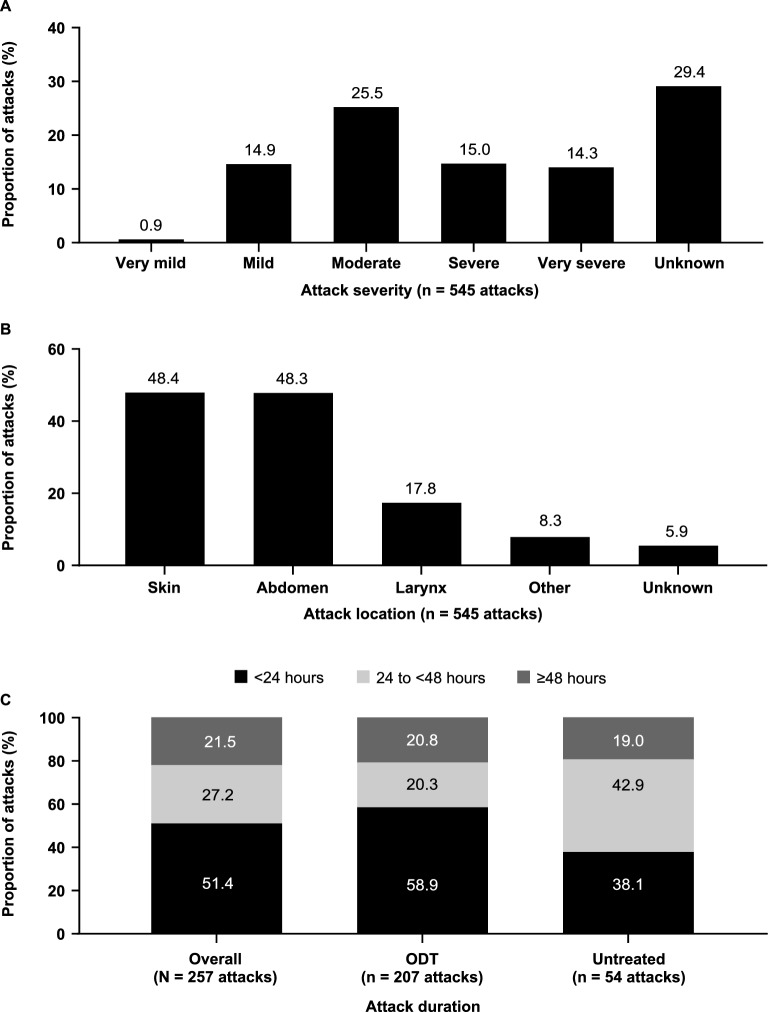
Characteristics of HAE attacks during the observation period. **A** Severity of HAE attacks before treatment. **B** Location of HAE attacks. An individual attack could have affected > 1 location. “Other” attack locations included joints, tongue, intestine, bladder, lungs, diaphragm, kidneys, and “mouth”. **C** Duration of attack symptoms (available for 257 attacks). *HAE* hereditary angioedema; *ODT* on-demand treatment

Data from 225 patients with HAE were included in the study and the patients were followed for a mean of 5.5 years; demographics are shown in Table 1. During the observation period, one patient died and the cause of death was deemed to be related to HAE (Additional file 1: Narrative S1). Data on 545 individual HAE attacks in 81 patients were available; for the remaining 144 patients, only attack frequency was reported, or attack information was not available (no data on individual attacks were available for the Italian cohort). Overall, 29.4% of attacks were considered severe or very severe (Fig. 1A) and 17.8% of attacks affected the larynx (Fig. 1B). Nearly half (48.6%) of 257 attacks lasted at least 24 h (Fig. 1C). Attacks treated with ODT resolved more quickly than untreated attacks (58.9% and 38.1% of treated and untreated attacks, respectively, lasted < 24 h). Ten attacks required an emergency procedure (n = 4 endotracheal intubation, n = 4 nasoendoscopy, n = 1 nasotracheal intubation, n = 1 unknown).

**Table 1:** Demographic and clinical characteristics, overall and by country

	Overall (N = 225)	US (n = 56)	Canada (n = 51)	Germany (n = 45)	Australia (n = 35)	UK (n = 24)	Italy (n = 14)
Follow-up duration (y), mean ± SD	5.5 ± 4.2	5.2 ± 4.0	7.0 ± 4.2	5.6 ± 3.4	5.9 ± 5.9	5.0 ± 2.3	1.3 ± 1.2
Year of index date, n (%)^a^							
2008 or earlier	37 (16.4)	5 (8.9)	17 (33.3)	9 (20.0)	6 (17.1)	0	0
2009–2011	52 (23.1)	20 (35.7)	9 (17.6)	8 (17.8)	8 (22.9)	7 (29.2)	0
2012–2014	56 (24.9)	8 (14.3)	19 (37.3)	13 (28.9)	7 (20.0)	6 (25.0)	3 (21.4)
2015–2017	69 (30.7)	18 (32.1)	6 (11.8)	13 (28.9)	12 (34.3)	10 (41.7)	10 (71.4)
2018 or later	11 (4.9)	5 (8.9)	0	2 (4.4)	2 (5.7)	1 (4.2)	1 (7.1)
Age at index date, mean ± SD^a^	36.8 ± 15.6	34.0 ± 12.9	35.7 ± 16.6	42.3 ± 15.8	32.1 ± 14.0	41.7 ± 17.6	38.0 ± 16.0
Female sex, n (%)	141 (62.7)	39 (69.6)	35 (68.6)	27 (60.0)	18 (51.4)	16 (66.7)	6 (42.9)
Race/ethnicity, n (%)							
White	194 (86.2)	52 (92.9)	51 (100.0)	27 (60.0)	32 (91.4)	22 (91.7)	10 (71.4)
Other^b^	7 (3.1)	4 (7.1)	0	0	3 (8.6)	0	0
Unknown	24 (10.7)	0	0	18 (40.0)	0	2 (8.3)	4 (28.6)
Type of HAE, n (%)							
Type 1	203 (90.2)	46 (82.1)	49 (96.1)	40 (88.9)	31 (88.6)	23 (95.8)	14 (100.0)
Type 2	22 (9.8)	10 (17.9)	2 (3.9)	5 (11.1)	4 (11.4)	1 (4.2)	0
Family history of HAE, n (%)							
Yes	160 (71.1)	46 (82.1)	33 (64.7)	27 (60.0)	24 (68.6)	20 (83.3)	10 (71.4)
No	38 (16.9)	8 (14.3)	13 (25.5)	8 (17.8)	2 (5.7)	3 (12.5)	4 (28.6)
Unknown	27 (12.0)	2 (3.6)	5 (9.8)	10 (22.2)	9 (25.7)	1 (4.2)	0
Comorbidities, (%)^c^							
Any comorbidities	105 (46.7)	29 (51.8)	34 (66.7)	12 (26.7)	17 (48.6)	9 (37.5)	4 (28.6)
Allergy or anaphylaxis^d^	36 (16.0)	8 (14.3)	18 (35.3)	2 (4.4)	7 (20.0)	0	1 (7.1)
Metabolic^e^	25 (11.1)	10 (17.9)	6 (11.8)	3 (6.7)	4 (11.4)	2 (8.3)	0
Psychiatric^f^	25 (11.1)	9 (16.1)	8 (15.7)	1 (2.2)	6 (17.1)	1 (4.2)	0
Cardiovascular^g^	22 (9.8)	5 (8.9)	5 (9.8)	4 (8.9)	4 (11.4)	3 (12.5)	1 (7.1)
Gastrointestinal^h^	20 (8.9)	2 (3.6)	13 (25.5)	0	3 (8.6)	1 (4.2)	1 (7.1)
Cancer^i^	9 (4.0)	0	6 (11.8)	1 (2.2)	1 (2.9)	1 (4.2)	0
Autoimmune^j^	3 (1.3)	0	1 (2.0)	2 (4.4)	0	0	0

*HAE* hereditary angioedema

^a^The index date is defined as the patient’s first HAE-related visit at the clinical center

^b^Other includes Asian, Black, Hispanic, and other

^c^Comorbidities occurring in > 5% overall are listed; malignancies and autoimmune disorders have a frequency < 5% but are also listed owing to particular interest in HAE and decreased complement levels

^d^Allergy or anaphylaxis included allergy, anaphylaxis, and allergic rhinitis

^e^Metabolic included obesity, diabetes, hypercalcemia, hyperthyroidism, dyslipidemia, hyperlipidemia, and hypothyroidism

^f^Psychiatric disorders included anxiety or depression, posttraumatic stress disorder, psychosis, attention deficit hyperactivity disorder, bipolar disorder, chronic schizophrenia, and disassociation personality disorder

^g^Cardiovascular included hypertension, cerebrovascular disease, hypercholesterolemia, myocardial infarction, congestive heart failure, peripheral vascular disease, pulmonary emboli, and cardiac arrhythmia

^h^Gastrointestinal included gastroesophageal reflux disease, hemorrhoid, irritable bowel syndrome, Schatzki’s ring, Crohn’s disease, diverticular disease, diverticulitis, peptic ulcer disease, ulcerative colitis, diverticulosis, inflammatory bowel syndrome, and gastric ulcer

^i^Cancer included prostate cancer, lymphoma, chronic lymphocytic leukemia, and adrenal myelolipoma

^j^Autoimmune included Graves’ disease, autoimmune thyroiditis, and immune defect

### Treatment patterns

ODT was prescribed to 193/225 (85.8%) patients during observation. The most common reasons for prescribing ODT were “timely access to care” (55.9%), “frequency of attacks” (51.7%), “history of laryngeal attacks” (20.7%), “emergency room (ER) visit or hospitalization due to HAE” (19.8%), and “rapid progression of attacks” (17.1%). On-demand treatment was used for a total of 421 (77.2%) attacks during the observation period. Of these, plasma-derived C1-INH was the most commonly used ODT (308 (73.2%) attacks, including 61.0% Berinert^®^ and 12.1% Cinryze^®^), followed by icatibant (99 (23.5%); Table 2). Danazol (mean (SD) dose 280 (192.4) mg) was used for five (0.9%) unique attacks experienced by five patients; two patients used a second dose of danazol to treat the same attack (dose 150 (70.7) mg). Stanozolol was also used by two patients (7.0 (1.4) mg dose) to treat an attack.

**Table 2:** On-demand treatments used for unique attacks, overall and by country

n (%)	Overall (N = 545)	US (n = 140)	Canada (n = 112)	Germany (n = 74)	Australia (n = 103)	UK (n = 116)
Yes	421 (77.2)	112 (80.0)	58 (51.8)	45 (60.8)	90 (87.4)	116 (100)
C1-INH: Berinert^®^	257 (61.0)	20 (17.9)	55 (94.8)	42 (93.3)	52 (57.8)	88 (75.9)
Intravenous	249 (96.9)	20 (100)	47 (85.5)	42 (100)	52 (100)	88 (100)
Subcutaneous^a^	8 (3.1)	0	8 (14.5)	0	0	0
Icatibant	99 (23.5)	60 (53.6)	1 (1.7)	3 (6.7)	33 (36.7)	2 (1.7)
C1-INH: Cinryze^®^	51 (12.1)	24 (21.4)	0	0	1 (1.1)	26 (22.4)
Androgens	7 (1.7)	2 (1.8)	2 (3.4)	0	3 (3.3)	0
Ecallantide	4 (1.0)	4 (3.6)	0	0	0	0
Fresh frozen plasma	3 (0.7)	2 (1.8)	0	0	1 (1.1)	0
No	54 (9.9)	11 (7.9)	6 (5.4)	27 (36.5)	10 (9.7)	0
Unknown	70 (12.8)	17 (12.1)	48 (42.9)	2 (2.7)	3 (2.9)	0

*C1-INH* C1 inhibitor, *HAE* hereditary angioedema

^a^Berinert is approved for intravenous administration to treat acute attacks of HAE in pediatric and adult patients. Subcutaneous administration for on-demand treatment of attacks is unapproved

Overall, 54/225 (24.0%) patients had at least one prescription for STP during follow-up. Intravenous Berinert^®^ was prescribed to 26 (48.1%) patients and danazol was prescribed to 14 (25.9%). The most common reasons for prescribing STP were dental and surgical procedures (35.6% and 32.9%, respectively).

Just over half of all patients (121/225 (53.8%)) received at least one prescription for LTP during the observation period (Table 3). The most common reasons for physicians to recommend LTP were “frequency of attacks” (70.8%), “impacts lifestyle (vacation, family, participation in sports)” (40.6%), “ER visit or hospitalization due to HAE” (27.1%), “history of laryngeal attacks” (25.5%), “rapid progression of attacks” (20.8%), and “missed days of school/work” (18.8%). The proportion of patients using LTP varied across countries, from 87.5% of patients in the United Kingdom to 7.1% in Italy.

**Table 3:** LTP treatment prescriptions, overall and by country

n (%)	Overall (N = 225)	US (n = 56)	Canada (n = 51)	Germany (n = 45)	Australia (n = 35)	UK (n = 24)	Italy (n = 14)
Patients with any LTP use^a^	121 (53.8)	34 (60.7)	33 (64.7)	13 (28.9)	19 (54.3)	21 (87.5)	1 (7.1)
Plasma-derived C1-INH							
Berinert^®^	31 (25.6)	2 (5.9)	17 (51.5)	4 (30.8)	7 (36.8)	1 (4.8)	0
Intravenous	25 (20.7)	2 (5.9)	14 (42.4)	1 (7.7)	7 (36.8)	1 (4.8)	0
Subcutaneous^b^	8 (6.6)	0	4 (12.1)	4 (30.8)	0	0	0
Cinryze^®^	36 (29.8)	21 (61.8)	6 (18.2)	7 (53.8)	0	2 (9.5)	0
Haegarda^®^	9 (7.4)	9 (26.5)	0	0	0	0	0
Androgen therapy							
Danazol	65 (53.7)	15 (44.1)	19 (57.6)	2 (15.4)	11 (57.9)	18 (85.7)	0
Stanozolol	7 (5.8)	3 (8.8)	1 (3.0)	0	3 (15.8)	0	0
Oxandrolone	3 (2.5)	0	0	0	1 (5.3)	2 (9.5)	0
Tranexamic acid	15 (12.4)	0	5 (15.2)	0	7 (36.8)	2 (9.5)	1 (100.0)
Ruconest^®^	1 (0.8)	1 (2.9)	0	0	0	0	0
Lanadelumab	11 (9.1)	7 (20.6)	0	4 (30.8)	0	0	0

Values are n (%)

*C1-INH* C1 inhibitor, *HAE* hereditary angioedema, *LTP* long-term prophylaxis

^a^Patients may have received ≥ 1 treatment prescription during follow-up

^b^Berinert^®^ is approved for intravenous administration to treat acute attacks of HAE in pediatric and adult patients. Subcutaneous administration for on-demand treatment of attacks is unapproved

During the observation period, androgens were the most commonly prescribed type of LTP; danazol, stanozolol, and oxandrolone were prescribed for LTP to 53.7%, 5.8%, and 2.5% of patients, respectively. Danazol use was highest in the United Kingdom (18/21 (85.7%) patients prescribed LTP) and lowest in Germany (2/13 (15.4%)); it was used by 15/34 (44.1%) of patients in the United States, and was not used in Italy. Cinryze^®^ and Berinert^®^ were prescribed to 29.8% and 20.7% of patients, respectively, and tranexamic acid was prescribed to 12.4% of patients for LTP.

Dosing was modified in 42/121 (34.7%) patients who were prescribed LTP, with 30 (24.8%) patients requiring dose up-titration (Table 4). Of 65 patients treated with danazol, 32.3% required dose modification, including 20% who required up-dosing. Of patients treated with Berinert^®^, Cinryze^®^, or tranexamic acid, 25.8%, 25%, and 20% of patients, respectively, required up-dosing. For all of these treatments, “failure to achieve satisfactory response” was the most common reason for up-dosing.

**Table 4:** Dose modifications among patients receiving any LTP treatment

	Overall (N = 121)	US (n = 34)	Canada (n = 33)	Germany (n = 13)	Australia (n = 19)	UK (n = 21)	Italy (n = 1)
Patients with any dose modification	42 (34.7)	15 (44.1)	13 (39.4)	2 (15.4)	4 (21.1)	8 (38.1)	0
Patients with any up-dosing^a^	30 (24.8)	10 (29.4)	13 (39.4)	0	2 (10.5)	5 (23.8)	0
Berinert^®^	n = 31	n = 2	n = 17	n = 4	n = 7	n = 1	n = 0
Dose modification	8 (25.8)	0	6 (35.3)	0	2 (28.6)	0	NA
Up-dosing	8 (25.8)	0	6 (35.3)	0	2 (28.6)	0	NA
Cinryze^®^	n = 36	n = 21	n = 6	n = 7	n = 0	n = 2	n = 0
Dose modification	13 (36.1)	12 (57.1)	1 (16.7)	0	NA	0	NA
Up-dosing	9 (25.0)	8 (38.1)	1 (16.7)	0	NA	0	NA
Danazol	n = 65	n = 15	n = 19	n = 2	n = 11	n = 18	n = 0
Dose modification	21 (32.3)	4 (26.7)	6 (31.6)	1 (50.0)	2 (18.2)	8 (44.4)	NA
Up-dosing	13 (20.0)	2 (13.3)	6 (31.6)	0	0	5 (27.8)	NA
Tranexamic acid	n = 15	n = 0	n = 5	n = 0	n = 7	n = 2	n = 1
Dose modification	3 (20.0)	NA	3 (60.0)	NA	0	0	0
Up-dosing	3 (20.0)	NA	3 (60.0)	NA	0	0	0
Lanadelumab	n = 11	n = 7	n = 0	n = 4	n = 0	n = 0	n = 0
Dose modification	1 (9.1)	0	NA	1 (25.0)	NA	NA	NA
Up-dosing	0	NA	0	0	NA	NA	NA

Percentages were calculated on the basis of number of patients using each type of LTP

*LTP* long-term prophylaxis, *NA* not applicable

^a^Up-dosing includes increased dose and increased dosing frequency

### LTP reduces HAE attack rate

Data on the frequency of HAE attacks were available from 131 patients (Table 5). The mean (SD) rate of HAE attacks was 20.2 (16.9) per year, ranging from 17.4 (17.0) in Canada to 25.6 (13.5) in Germany. Of the 131 patients, 79 (60.3%) received at least one prescription for LTP during the period(s) in which attack frequency was reported.

**Table 5:** HAE attack rates during follow-up, overall and by country

	Overall (N = 131)	US (n = 34)	Canada (n = 42)	Germany (n = 17)	Australia (n = 19)	UK (n = 12)	Italy (n = 7)
Annual attack rate							
Mean ± SD	20.2 ± 16.9	21.8 ± 17.5	17.4 ± 17.0	25.6 ± 13.5	17.8 ± 14.8	23.4 ± 24.7	17.5 ± 9.7
Median (range)	14.1 (0, 84.1)	15.9 (0, 72.0)	11.9 (0.3, 79.3)	24.0 (4.0, 52.2)	14.0 (2.0, 52.2)	11.8 (0.3, 84.1)	13.8 (6.0, 33.2)
Received LTP^a^	79 (60.3)	22 (64.7)	28 (66.7)	6 (35.3)	11 (57.9)	11 (91.7)	1 (14.3)
Annual attack rate							
Mean ± SD	20.8 ± 18.9	26.9 ± 19.1	14.6 ± 13.2	35.4 ± 13.8	12.9 ± 15.2	25.0 ± 28.3	9.7 ±
Median (range)	13.9 (0, 84.1)	20.7 (0, 72.0)	11.6 (0.3, 52.2)	30.0 (24.0, 52.2)	5.6 (1.1, 52.2)	12.0 (0.3, 84.1)	9.7 (9.7, 9.7)
Did not receive LTP^a^	52 (39.7)	12 (35.3)	14 (33.3)	11 (64.7)	8 (42.1)	1 (8.3)	6 (85.7)
Annual attack rate							
Mean ± SD	20.3 ± 16.8	14.1 ± 14.7	23.4 ± 24.4	20.2 ± 10.3	22.9 ± 14.3	43.0 ±	18.7 ± 10.1
Median (range)	15.7 (0.3, 79.3)	9.4 (0.9, 48.2)	15.7 (0.3, 79.3)	24.0 (4.0, 36.0)	23.8 (2.0, 52.2)	43.0 (43.0, 43.0)	18.9 (6.0, 33.2)

Only patients with information on frequency of HAE attacks are included

*HAE* hereditary angioedema, *LTP* long-term prophylaxis, *SD* standard deviation

^a^LTP was or was not received during the period(s) in which frequency of HAE attacks was reported

After adjusting for patient characteristics representing disease activity (HAE attacks before index date) and other baseline covariates, patients who received any LTP treatment prescriptions had a statistically significantly lower rate of HAE attacks compared with those who did not receive LTP (incidence rate ratio (95% CI) 0.90 (0.84–0.96); *P* = 0.002) (Table 6).

**Table 6:** Multivariable Poisson regression analysis of HAE attack rate during follow-up

	HAE attack rate (N = 131)	
	IRR (95% CI)	*P*-value
Treatment type		
Any LTP (ref: no LTP)	0.90 (0.84–0.96)	0.002
Baseline covariates		
Male sex (ref: female)	0.94 (0.88–1.00)	0.043
Age at HAE diagnosis (per 10 years)	1.03 (1.01–1.05)	0.001
HAE attacks before index date (ref: < median)	1.67 (1.57–1.77)	< 0.001
Country of residence (ref: US)		
Canada	0.76 (0.70–0.81)	< 0.001
Germany	0.91 (0.83–1.01)	0.075
Australia	0.85 (0.78–0.92)	< 0.001
UK	0.86 (0.76–0.97)	0.014
Italy	0.48 (0.37–0.63)	< 0.001
Family history of HAE (ref: no family history)	1.27 (1.17–1.37)	< 0.001
Type 1 HAE (ref: type 2 HAE)	0.81 (0.75–0.88)	< 0.001
Any preexisting comorbidity (ref: no preexisting comorbidity)	1.13 (1.07–1.20)	< 0.001
Previous misdiagnosis (ref: no previous misdiagnosis)	0.83 (0.75–0.91)	< 0.001

*CI* confidence interval, *HAE* hereditary angioedema, *IRR* incidence rate ratio, *LTP* long-term prophylaxis

### LTP discontinuation

During observation, LTP treatments were discontinued in 76 treatment periods. The most common reasons were “intolerable adverse drug reaction(s)” (28.9%), “medication not effective” (22.4%), “discontinued access to medication” (13.2%), “inconvenient route of administration” (11.8%), and “fear of potential adverse drug reaction(s)” (10.5%) (Table 7).

**Table 7:** Reasons for discontinuation of LTP

	Overall (N = 225)	US (n = 56)	Canada (n = 51)	Germany (n = 45)	Australia (n = 35)	UK (n = 24)	Italy (n = 14)
Patients who discontinued LTP	n = 76	n = 26	n = 25	n = 4	n = 15	n = 6	n = 0
Intolerable adverse drug reaction(s)	22 (28.9)	4 (15.4)	7 (28.0)	0	7 (46.7)	4 (66.7)	–
Medication not effective	17 (22.4)	3 (11.5)	8 (32.0)	1 (25.0)	5 (33.3)	0	–
Discontinued access to medication	10 (13.2)	6 (23.1)	1 (4.0)	0	3 (20.0)	0	–
Inconvenient route of administration	9 (11.8)	6 (23.1)	3 (12.0)	0	0	0	–
Fear of potential adverse drug reaction(s)	8 (10.5)	1 (3.8)	7 (28.0)	0	0	0	–
Patient noncompliance	3 (3.9)	0	2 (8.0)	0	1 (6.7)	0	–
Drug–drug interactions	2 (2.6)	0	0	2 (50.0)	0	0	–
Drug–concomitant disease interactions	2 (2.6)	0	0	0	2 (13.3)	0	–
Other^a^	17 (22.4)	4 (15.4)	10 (40.0)	1 (25.0)	1 (6.7)	1 (16.7)	–
Unknown	7 (9.2)	4 (15.4)	1 (4.0)	1 (25.0)	0	1 (16.7)	–
Berinert^®^	n = 9	n = 2	n = 6	n = 1	n = 0	n = 0	–
Inconvenient route of administration	2 (22.2)	0	2 (33.3)	0	–	–	–
Medication not effective	1 (11.1)	1 (50.0)	0	0	–	–	–
Patient noncompliance	1 (11.1)	0	1 (16.7)	0	–	–	–
Other^a^	5 (55.6)	1 (50.0)	3 (50.0)	1 (100)	–	–	–
Cinryze^®^	n = 17	n = 15	n = 1	n = 1	n = 0	n = 0	–
Discontinued access to medication	6 (35.3)	6 (40.0)	0	0	–	–	–
Inconvenient route of administration	6 (35.3)	6 (40.0)	0	0	–	–	–
Medication not effective	3 (17.6)	2 (13.3)	1 (100)	0	–	–	–
Other^a^	1 (5.9)	1 (6.7)	0	0	–	–	–
Unknown	3 (17.6)	2 (13.3)	0	1 (100)	–	–	–
Danazol	n = 32	n = 6	n = 12	n = 2	n = 8	n = 4	–
Intolerable adverse drug reaction(s)	17 (53.1)	3 (50.0)	6 (50.0)	0	5 (62.5)	3 (75.0)	–
Fear of potential adverse drug reaction(s)	7 (21.9)	1 (16.7)	6 (50.0)	0	0	0	–
Medication not effective	5 (15.6)	0	3 (25.0)	1 (50.0)	1 (12.5)	0	–
Drug–drug interaction(s)	2 (6.3)	0	0	2 (100)	0	0	–
Discontinued access to medication	2 (6.3)	0	0	0	2 (25.0)	0	–
Inconvenient route of administration	1 (3.1)	0	1 (8.3)	0	0	0	–
Drug–concomitant disease interaction(s)	1 (3.1)	0	0	0	1 (12.5)	0	–
Other^a^	9 (28.1)	1 (16.7)	7 (58.3)	0	1 (12.5)	0	–
Unknown	3 (9.4)	1 (16.7)	1 (8.3)	0	0	1 (25.0)	–
Stanozolol	n = 6	n = 2	n = 1	n = 0	n = 3	n = 0	–
Intolerable adverse drug reaction(s)	2 (33.3)	1 (50.0)	0	–	1 (33.3)	–	–
Discontinued access to medication	2 (33.3)	0	1 (100)	–	1 (33.3)	–	–
Medication not effective	1 (16.7)	0	0	–	1 (33.3)	–	–
Drug–concomitant disease interaction(s)	1 (16.7)	0	0	–	1 (33.3)	–	–
Unknown	1 (16.7)	1 (50.0)	0	–	0	–	–
Oxandrolone	n = 1	n = 0	n = 0	n = 0	n = 1	n = 0	–
Medication not effective	1 (100)	–	–	–	1 (100)	–	–
Tranexamic acid	n = 10	n = 0	n = 5	n = 0	n = 3	n = 2	–
Medication not effective	6 (60.0)	–	4 (80.0)	–	2 (66.7)	0	–
Intolerable adverse drug reaction(s)	3 (30.0)	–	1 (20.0)	–	1 (33.3)	1 (50.0)	–
Patient noncompliance	2 (20.0)	–	1 (20.0)	–	1 (33.3)	0	–
Fear of potential adverse drug reaction(s)	1 (10.0)	–	1 (20.0)	–	0	0	–
Other^a^	1 (10.0)	–	0	–	0	1 (50.0)	–
Ruconest^®^	n = 1	n = 1	n = 0	n = 0	n = 0	n = 0	–
Other^a^	1 (100)	1 (100)	–	–	–	–	–

The prescribed LTP treatment was available for 121 patients accounting for 192 treatment periods. A gap of 30 days between treatment prescriptions or a treatment interruption of > 30 days was considered a new treatment period

The abstractor was asked to provide reasons for discontinuation of treatment, choosing from a list of common factors, free text entry, or “unknown”. ≥ 1 response may have been selected

*LTP* long-term prophylaxis

^a^“Other” includes factors such as cancer, patient preference, self-administration of Berinert^®^, patient having fewer attacks, patient went onto study, and pregnancy

Androgens were the most commonly discontinued treatment (51.3%), with poor tolerability and fear of potential adverse events cited as the reason for discontinuation in 53.1% and 21.9% of patients, respectively. Intravenous plasma-derived C1-INH therapy accounted for 34.2% of discontinuations, with inconvenient route of administration cited as the reason in 31.8% of patients. Tranexamic acid therapy made up 13.2% of discontinuations, with 60% of patients citing lack of effectiveness.

### Healthcare resource utilization

Information on HRU was available from 137/225 (60.9%) patients; most patients (128 (93.4%)) had routine HAE-related outpatient visits (Table 8). However, 70/137 (51.1%) patients had one or more HAE attack-related HRU visits: 38.7% visited the ER owing to an HAE attack, 8.8% were hospitalized, and 23.4% made outpatient visits. Data on HRU visits in Germany were unavailable.

**Table 8:** HAE-related HRU, overall and by country

Patients with any HRU episodes, N (%) Annual rate, mean ± SD [median]	Overall (N = 225)	US (n = 56)	Canada (n = 51)	Germany (n = 45)	Australia (n = 35)	UK (n = 24)	Italy (n = 14)
HRU visits (any reason)	137 (60.9) 4.4 ± 9.0 [2.2]	23 (41.1) 3.5 ± 4.4 [2.1]	50 (98.0) 4.3 ± 12.0 [2.2]	N/A	34 (97.1) 5.9 ± 9.1 [2.5]	24 (100) 4.0 ± 4.5 [2.6]	6 (42.9) 1.8 ± 1.8 [1.1]
Routine HAE-related outpatient visits	128 (93.4) 2.3 ± 4.2 [1.6]	21 (91.3) 1.7 ± 2.2 [1.0]	45 (90.0) 1.8 ± 1.1 [1.5]	N/A	33 (97.1) 3.4 ± 7.4 [1.6]	24 (100) 2.6 ± 2.8 [1.9]	5 (83.3) 1.5 ± 1.9 [0.7]
HAE attack-related visits	70 (51.1) 2.8 ± 10.2 [0.7]	12 (52.2) 0.8 ± 1.1 [0.4]	30 (60.0) 3.9 ± 15.2 [0.8]	N/A	19 (55.9) 2.3 ± 3.8 [1.0]	8 (33.3) 2.7 ± 3.4 [0.7]	1 (16.7) 2.9 ± [2.9]
Emergency room visits	53 (38.7) 1.3 ± 1.8 [0.7]	8 (34.8) 0.6 ± 0.7 [0.2]	23 (46.0) 1.0 ± 1.2 [0.8]	N/A	17 (50.0) 1.9 ± 2.4 [0.7]	5 (20.8) 2.1 ± 2.7 [0.7]	0 NA
Hospitalizations	12 (8.8) 1.1 ± 2.3 [0.3]	0 NA	5 (10.0) 0.2 ± 0.1 [0.1]	N/A	4 (11.8) 2.4 ± 3.8 [0.6]	3 (12.5) 1.0 ± 1.2 [0.4]	0 NA
Outpatient visits^a^	32 (23.4) 3.5 ± 14.8 [0.3]	7 (30.4) 0.7 ± 1.3 [0.2]	15 (30.0) 6.3 ± 21.6 [0.3]	N/A	4 (11.8) 0.4 ± 0.4 [0.4]	5 (20.8) 1.7 ± 2.3 [0.7]	1 (16.7) 2.9 ± [2.9]

*HAE* hereditary angioedema, *HRU* healthcare resource utilization, *NA* not applicable, N*/*A not available

^a^Outpatient visits included physician visits and other outpatient visits

## Discussion

This is the largest chart review study published to date of patients with HAE describing real-world clinical practice. The study included a large number of patients, and data were collected across six developed countries, providing a detailed global snapshot of HAE disease characteristics and management at the time of data collection, including treatment patterns, outcomes, and HRU.

HAE attacks were frequent, with an overall mean (SD) attack rate of 20.2 (16.9) per year, which is slightly lower than the rate of 26.9 (43.1) attacks per year reported in a study conducted by Wilson et al. [19] approximately 10 years before our study period. In that study, 16% of 457 patients surveyed had visited an ER with regard to their most recent attack; the most common treatment administered for an attack was androgens (36.3%), compared with 5.7% of patients who received C1-INH. Furthermore, 49.5% of patients used androgens for LTP, compared with 14.2% who used C1-INH, and 0.4% who used tranexamic acid [19]. In the current study, approximately half of all patients with available data required care due to an attack, including almost 10% who were hospitalized. Use of androgens for LTP remained high in the United States (44.1% and 8.8% of patients were prescribed danazol and stanozolol, respectively) but there is also substantial use of C1-INH (61.8% Cinryze^®^, 5.9% Berinert^®^, 26.5% Haegarda^®^). These findings suggest that although attack rates in the current international study decreased since the US-based Wilson et al. study, likely due to improved HAE therapies, patients still experienced substantial disease burden and that HAE may not be adequately controlled by currently available treatment options [20].

Despite guidelines recommending that all patients carry ODT in case of an attack and that all attacks be treated as early as possible to minimize progression and potential mortality [2, 20, 21], only approximately 86% of patients received a prescription for ODT, and about 10% of attacks were not treated. Notably, some patients used androgens to treat attacks even though they are not indicated for on-demand use [2], indicating a need for more education on appropriate medications for treating an attack. However, the unavailability and higher cost of approved on-demand treatments, as well as the relative ease of access to androgens, may also have contributed to the use of androgens for this purpose.

In addition to ODT, consideration of LTP therapy is strongly recommended to prevent attacks [2, 20, 21]. After adjusting for patient characteristics representing disease activity and other confounders, the attack rate in this study was lower in patients who received LTP treatment compared with those who did not. However, there are still gaps in LTP use: just over half of all patients had a prescription for LTP treatment during the follow-up period, and the proportion of patients who used LTP and type of LTP prescribed varied across countries. These differences could be attributed to product availability at the time of chart abstraction and health systems that influence prescribing practices. For example, in Australia, coverage for the cost of prophylaxis with C1-INH is restricted to patients who experience eight or more attacks per month [22]; and in the United Kingdom, the recommended first line of treatment is androgens or antifibrinolytics, followed by consideration for C1-INH for patients who continue to experience at least two attacks per week [23]. Individual public and private insurers in the United States also have conditions and restrictions for coverage of specific medications.

While varied, notable use of androgens as LTP was observed at the time of this chart review, although several HAE guidelines do not recommend them as first line-therapy [2, 20, 21]. Androgens may be effective and tolerated in some patients with lower disease activity using lower doses [7], and patients for whom androgens provide a level of protection against attacks may prefer to continue with this convenient and inexpensive option over other available treatments. However, approximately half of patients on androgen therapy in this analysis discontinued treatment because of poor tolerability, consistent with a recent case report of 10 HAE patients who discontinued androgens, in which the most frequent reasons for discontinuation included the occurrence of side effects, insufficient control of attacks, and contraindications [24]. Dose up-titration was also required for 20% of patients on androgens in this study period. However, these prescription patterns, along with a persistently high attack rate despite LTP use, suggest that there remains a need for more effective LTP treatments.

Plasma-derived C1-INH, lanadelumab, and berotralstat are currently recommended as first-line LTP [21]. Although effective in preventing HAE attacks, C1-INH therapeutics require intravenous or subcutaneous injections every 3–4 days. In this study, inconvenient route of administration was cited as a frequent reason for discontinuation of Cinryze^®^ and off-label Berinert^®^. In addition, as these products are derived from donated human plasma, supply shortages can impact production and availability. Lanadelumab is a monoclonal antibody that is administered by subcutaneous injection every 2 weeks, with the option of reducing the frequency to every 4 weeks in some geographies if a patient is well controlled. However, adverse events associated with the injection site are frequent [15]. Berotralstat is a daily oral LTP and is the newest LTP product on the market. Although convenient, it is associated with drug interactions and gastrointestinal reactions [25]. Regulatory approval of these modern treatments has been limited mainly to developed countries thus far, with lanadelumab having been newer to the market and berotralstat not yet available at the time of this chart review. Even in regions where they are licensed, extremely high costs have led some payors to restrict access to only patients with severe disease and for whom androgens are ineffective, intolerable, or contraindicated [22, 23]. As a result, there is a treatment gap in some regions for patients for whom androgens are not a safe or effective option, but whose disease is not severe enough to qualify for coverage of newer, more expensive treatments.

One limitation of this study is that the reported rates of oral androgen prescribing with the purpose of long-term prophylaxis may be a result of combining data across all years included in this study (≤ 2008 to 2019) and does not reflect clinical practice over more recent years. In Canada, for example, an increase in the number of prescriptions for plasma-derived C1-INH LTP therapies, accompanied by a decline in prescriptions of oral androgen LTP therapies, was observed after 2014 [26, 27]. Similarly, a subgroup analysis of LTP use in Australian patients from the current dataset showed that 22.2% of patients were prescribed danazol after October 2016, compared with 57.9% of the overall Australian patient population during the entire follow-up period. This suggests a relatively recent move away from androgen use, however, discontinuation was still considerable with C1-INH and tranexamic acid therapies, further highlighting the need for effective LTP treatments.

Other limitations of this study include the incompleteness of some data records at the participating clinical centers; some data (for example, on individual HAE attacks and HRU) were not available at some centers, and data relating to HRU in an emergency setting may not always have been relayed accurately to the appropriate clinic center for recording in medical charts. This and the small sample size from Italy (n = 14) may have contributed to the apparent large differences in treatment patterns between patients in Italy and the other countries. In particular, data on individual attacks during the observation period were not available for the Italian cohort. It should also be noted that the Italian patients were all diagnosed within the last 5 years of chart abstraction. As a result, they may have had less severe disease that did not yet require LTP use.

Also, in common with all chart reviews, there is a risk that residual confounding variables may affect the analysis, despite the adjustments that were made using multivariable analyses. Analysis of the data by year may provide a better indication of how the management of HAE is affected by the availability of newly authorized treatments. Although patients received prescriptions for LTP, compliance with treatment is unknown and could have impacted the apparent effectiveness of the treatment. Finally, although this was an international study, data on patients of non-White race were limited (3.1% overall), and only developed countries were included, which may have introduced bias with respect to the availability and accessibility of LTP therapies. There would be value in conducting a similar study in developing countries.

Findings from this study add to the current knowledge of HAE treatment patterns and demonstrate an unmet need for more effective LTP treatment. Use of LTP has been shown to effectively prevent attacks; in agreement with current management guidelines, physicians should discuss the benefits of LTP with all patients. Although older therapies such as androgens provide adequate protection for some patients, newly available LTP treatments have been demonstrated to improve outcomes further, potentially leading to better quality of life for patients and reduced attack-related HRU. However, the availability and accessibility of these newer treatments may be limited, representing a major barrier to obtaining optimum treatment for some patients. A follow-up study would be beneficial to understand the impact of current guidelines and the availability of new therapies.

## Supplementary Information

**Additional file 1: Fig. S1.** Study design and patient eligibility criteria. **Narrative S1.** Patient death.

## Data Availability

The data that support the findings of this study are available from Analysis Group Inc. The datasets, including the redacted study protocol, redacted statistical analysis plan, and individual participants data supporting the results reported in this article, will be made available within 3 months from initial request, to researchers who provide a methodologically sound proposal. The data will be provided after their de-identification, in compliance with applicable privacy laws, data protection and requirements for consent and anonymization. Requests for data should be sent to DataSharing@Takeda.com.

## Abbreviations

C1-INH: C1 inhibitor
ER: Emergency room
HAE: Hereditary angioedema
HRU: Healthcare resource utilization
LTP: Long-term prophylaxis
ODT: On-demand treatment
SD: Standard deviation
STP: Short-term prophylaxis
