# Supplementary material for: A multicenter chart review of patient characteristics, treatment, and outcomes in hereditary angioedema: unmet need for more effective long-term prophylaxis
Source: Allergy Asthma Clin Immunol. 2023 May 29;19:48. doi: 10.1186/s13223-023-00795-2 (PMC10227962; doi:10.1186/s13223-023-00795-2)
Supplement: Supplementary file 1 — Additional file 1: Fig. S1. Study design and patient eligibility criteria. Narrative S1. Patient death. [file 13223_2023_795_MOESM1_ESM.docx]

**Additional information**

**Fig. S1** Study design and patient eligibility criteria


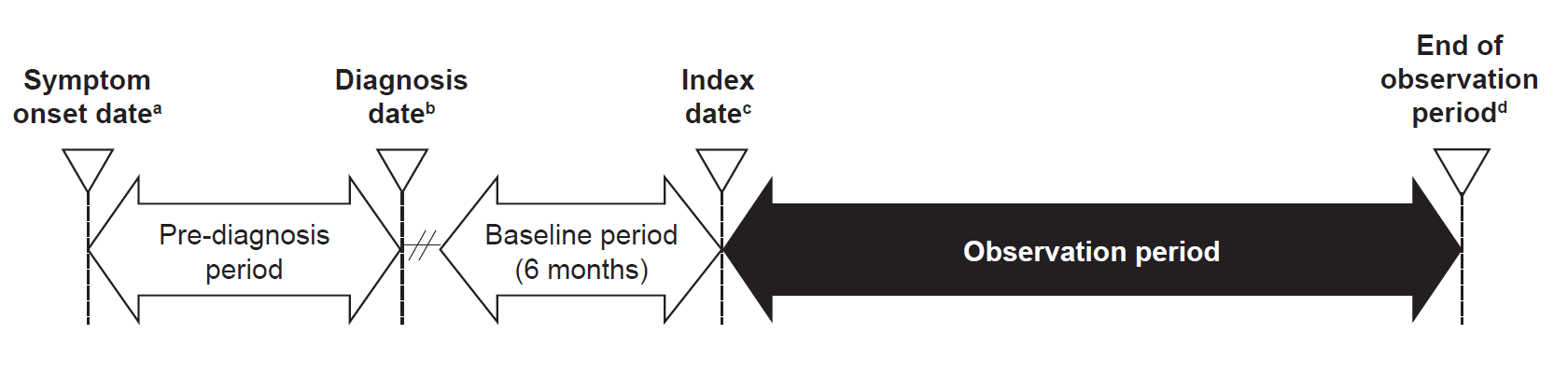


^a^Date of first symptom of hereditary angioedema (HAE).

^b^Date of documented diagnosis of HAE.

^c^Date of first HAE-related visit at participating clinical center.

^d^Earliest of last clinical visit, enrollment in a clinical trial for an investigational HAE treatment, death, or end of study.

**Narrative S1** Patient death

During the observation period, one patient died and the cause of death was deemed to be related to HAE. At the time of death, the patient was prescribed Berinert^®^ for LTP, as well as Berinert^®^ and icatibant for on-demand treatment. No other details regarding the death, including individual HAE attack information, were reported for this patient.
